# Efficient Electronic‐Structure Methods Toward Catalyst Screening: Projection‐Based Embedding Theory for CO_2_ Reduction Reaction Intermediates

**DOI:** 10.1002/anie.202503418

**Published:** 2025-08-07

**Authors:** Elena Kolodzeiski, Christopher J. Stein

**Affiliations:** ^1^ Department of Chemistry, TUM School of Natural Sciences Technical University of Munich Garching Germany; ^2^ Catalysis Research Center Technical University of Munich Garching Germany; ^3^ Atomistic Modeling Center Technical University of Munich Garching Germany

**Keywords:** CO_2_ conversion, Heterogeneous catalysis, Quantum‐chemical calculations, Quantum embedding, Reactivity studies

## Abstract

Catalyst screening is a demanding task for computational chemistry since the profound diversity of surface structures under *operando* conditions is accompanied by high demands on the accuracy to predict the relevant kinetics. Embedding approaches that allow researchers to focus the computational effort on the chemically active regions of interest are promising tools in the pursuit of balancing accuracy and efficiency. However, for metallic catalysts, the required separation of the system into an active part treated with highly accurate methods and an environment is technically hard to achieve due to the delocalization of electrons in the conducting surface. Therefore, studies analyzing the potential of embedding methods for heterogeneous (electro‐)catalyst screening are scarce. In this contribution, we demonstrate that simple embedding approaches are indeed achievable for studying metallic catalysts if i) the active orbital space is held consistent over a reaction coordinate and ii) the nonadditive exchange‐correlation functional used to calculate the embedding potential includes a fraction of exact exchange to mitigate delocalization errors. We verify the approach for a set of open‐ and closed‐shell CO_2_ reduction reaction intermediates on different adsorption sites of a Cu(111) surface represented by cluster models to demonstrate that catalyst screening with embedding approaches is achievable.

## Graphical abstract

This work shows how projection‐based embedding theory can be successfully applied to study chemical reactions on metal surfaces by providing guidelines for adequate system partitioning and an appropriate description of the intersystem interactions. This efficient and systematically improvable approach contributes significantly to the computational chemistry toolbox for heterogeneous catalysis as it paves the way toward routine application.

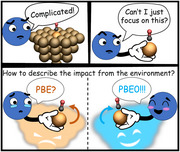

## Introduction

The conversion of carbon dioxide into valuable products has gained significant attention as it offers a pathway to mitigate greenhouse gas emissions.^[^ ^1^, ^2^ ^]^ In this regard, copper surfaces are among the most promising catalysts for facilitating the conversion of CO_2_, since they have been shown to yield valuable products with more than a single carbon.^[^ ^2^, ^3^, ^4^ ^]^ However, despite the immense interest, improving the selectivity and efficiency of these catalysts remains a critical challenge, as rational advancements require a thorough understanding of the underlying reaction mechanisms.^[^ ^5^, ^6^ ^]^ Therefore, computational modeling plays a crucial role, as it offers accurate insights into structural and electronic properties of short‐lived reaction intermediates adsorbed on the surface, which are elusive to most experimental methods.^[^ ^7^, ^8^ ^]^

Periodic density functional theory (DFT) is one of the most widely used theoretical methods in respective computational studies.^[^ ^9^, ^10^, ^11^ ^]^ It has shown remarkable success during the last decades, not only for reproducing experimental findings but also for predicting properties, which is highly important for material design.^[^ ^12^, ^13^, ^14^ ^]^ Despite these significant achievements, this approach suffers from the required approximation of the exchange‐correlation functional,^[^ ^15^ ^]^ which generally leads to unpredictable errors. The calculation of adsorption energies, activation barriers, and most favorable interaction sites is therefore hampered by the absence of systematically convergent computational protocols. One famous and controversially discussed example of this failure is the description of the adsorption mechanism of carbon monoxide (CO) on Cu(111).^[^ ^16^, ^17^ ^]^ When using semi‐local exchange‐correlation functionals such as PBE, DFT fails to predict the preferred adsorption site.^[^ ^18^ ^]^ This problem is known as the CO adsorption puzzle, highlighting the demand for a systematically improvable approach.^[^ ^19^, ^20^, ^21^ ^]^ In contrast, modern computational methods based on wavefunction theory (WFT), such as configuration interaction,^[^ ^22^, ^23^ ^]^ coupled cluster,^[^ ^24^, ^25^ ^]^ or perturbative post‐Hartree–Fock (HF) methods,^[^ ^26^, ^27^ ^]^ meet this requirement and allow for highly accurate and systematically improvable calculations but at the price of high numerical costs. The application of WFT on metal surfaces is thus not possible in routine calculations due to the large number of electrons and the corresponding numerical demands.

However, many chemical processes in heterogeneous catalysis are predominately determined by changes within a localized environment.^[^ ^28^, ^29^, ^30^ ^]^ Cluster models have, therefore, gained emerging interest in the context of metal surface studies since they provide a proper platform for investigating local phenomena. These models are typically relatively small, which enables the consideration of more accurate methods for describing the chemically active region at moderate numerical costs.^[^ ^31^, ^32^ ^]^ Although cluster approaches promise improvements when studying local properties, nonlocal interactions and edge effects, originating from under‐coordinated atoms, are largely ignored. Further, cluster models exhibit discrete energy levels, which may include a sizeable band gap. When forming bonds between the surface and the adsorbate, the electronic structure of both fragments must change, which is only possible through mixing with excited states. While these excitations occur on metal surfaces around the Fermi level at almost zero energy cost, a finite excitation energy must be considered on cluster models to reach the bonding state.^[^ ^33^, ^34^, ^35^, ^36^, ^37^ ^]^ Such finite‐size effects can considerably compromise the accuracy of the calculations, leading to inaccuracies despite the usage of highly accurate methods. Hence, the cluster model and the electronic‐structure approach must be carefully balanced to allow for an accurate description of interactions while properly taking long‐range interactions from the (metallic) environment into account. Employing embedding methods can, in this regard, be a remedy regarding the long‐range interactions. These methods introduce a balanced compromise between efficiency and accuracy, as they reduce the computational costs of quantum‐chemical calculations by separating the system into an active subsystem, where the structural and electronic changes occur, and an environment.^[^ ^38^, ^39^, ^40^ ^]^ The active subsystem is the focus of the computational efforts and is thus treated using an accurate and computationally demanding method, while the environment is calculated using an efficient but less accurate method. Versatile quantum embedding schemes such as QM/MM,^[^ ^41^ ^]^ implicit solvents,^[^ ^42^, ^43^ ^]^ ONIOM models, inclusively electrostatic embedding,^[^ ^44^, ^45^ ^]^ density matrix embedding theory,^[^ ^46^ ^]^ Green's function embedding,^[^ ^47^, ^48^, ^49^ ^]^ frozen density embedding, and subsystem DFT,^[^ ^50^, ^51^, ^52^, ^53^, ^54^, ^55^, ^56^ ^]^ among many others,^[^ ^57^, ^58^, ^59^ ^]^ have been developed. However, the embedding of localized clusters within a periodic environment still exhibits a serious challenge, e.g., due to the different representations of the wave function.^[^ ^40^, ^60^ ^]^ Transformations from Gaussian basis to periodic wave functions are not trivial and frequently require the interfacing of several program packages. For the sake of simplicity, many embedding studies focus, therefore, on nonperiodic embedding schemes.

Projection‐based embedding theory (PBET) is a subsystem DFT approach offering a robust, accurate, and simple framework for DFT‐in‐DFT and even WF‐in‐DFT embedding.^[^ ^55^, ^56^ ^]^ Although PBET is designed for applications for numerically demanding systems, studies dealing with strongly correlated many‐electron systems such as metal nanoclusters or surfaces are limited.^[^ ^61^, ^62^, ^63^ ^]^ Most reported studies focus on isolated organic systems or transition‐metal complexes.^[^ ^64^, ^65^, ^66^, ^67^, ^68^, ^69^ ^]^ The success of a PBET calculation depends on the proper partitioning of the active and environmental subsystems and an accurate description of their interactions. However, the electronic structure of organic and metallic systems differs significantly. PBET aggressively partitions both subsystems, even across covalent bonds. This gets more complicated when applied to metal surfaces exhibiting strongly correlated and nonlocal electron–electron interactions. Consequently, its accuracy, which has been repeatedly demonstrated for organic systems and transition metal complexes,^[^ ^40^, ^70^, ^71^, ^72^, ^73^ ^]^ cannot be taken for granted in the case of metal surfaces. Systematic studies that analyze the effects of different system partitioning and different descriptions of the intersystem interactions are essential, as these factors are the primary sources of error within the PBET framework.^[^ ^30^, ^40^ ^]^ Understanding and addressing these properties will significantly enhance the accuracy and reliability.

From our previous study,^[^ ^62^ ^]^ it is already known that conventional partitioning schemes based on orbital localization procedures such as Pipek–Mezey^[^ ^74^ ^]^ (PM) or intrinsic bond orbitals^[^ ^75^ ^]^ (IBOs) are error‐prone when applied to challenging systems such as metal clusters with many delocalized and (near‐) degenerate molecular orbitals.^[^ ^62^ ^]^ For these systems, active space selection based on the SPADE algorithm has proven to be more suitable.^[^ ^62^ ^]^ In contrast to conventional selection schemes, SPADE tends to preserve the delocalized nature of the electronic structure within the respective subsystem.^[^ ^62^, ^76^ ^]^ The initial SPADE algorithm invented by Claudino and Mayhall is a parameter‐free selection scheme based on a singular value decomposition applied to the active subsystem.^[^ ^76^ ^]^ The largest gap between consecutive singular values defines the most suitable system partitioning. When energy differences are to be calculated, keeping the orbital space consistent for all structures is pivotal. However, this is not ensured a priori by applying the SPADE algorithm since the position of the largest gap depends on the molecular structure and might change along reaction pathways. Therefore, most recently, we developed the ACE‐of‐SPADE algorithm, which enables a consistent active orbital space selection, even for altering molecular structures on metal nanoclusters.^[^ ^62^ ^]^

Apart from the system partitioning, another potential source of errors is concealed in the description of the inter‐subsystem coupling, which depends on the availability of an accurate description of the environment and of the subsystem's interaction with it.^[^ ^30^, ^77^ ^]^ In periodic DFT, metal surfaces are usually described using semi‐local generalized gradient approximation (GGA) functionals.^[^ ^78^, ^79^ ^]^ These functionals promote the delocalization of molecular orbitals, making them a reliable choice for modeling the metallic environment in PBET. While GGA functionals effectively capture important properties of bulk metals, they are prone to delocalization errors when local bonds are to be described.^[^ ^80^ ^]^ The delocalized electrons from the bulk may spill over onto locally formed bonds, resulting in overstabilization, which in turn leads to overestimation of binding energies of organic molecules adsorbed on the metal surface.^[^ ^81^, ^82^, ^83^ ^]^ Although the active subsystem is treated at a higher level of theory—potentially including exact exchange, which helps mitigating delocalization errors^[^ ^80^ ^]^—it is still unclear how the delocalized orbitals from the environment and the corresponding intersystem interaction affect the local bond formation in the active region.

Note, significant errors due to the band gap of the embedded cluster models are not to be expected. The projection‐based embedding theory considers the total energy of the system in terms of a correction scheme based on the energy difference of the embedded cluster at high and low levels of theory. Consequently, errors originating from the cluster's geometry cancel out (see Supporting Information for further computational details).^[^ ^32^ ^]^

In addition to classical Coulombic interactions, the interaction between both subsystems is described by a nonadditive exchange‐correlation functional. Therefore, mixing fractions of exact exchange into this functional also affects the delocalization errors in the active subsystem. For reliable investigations of adsorbate‐surface interactions using PBET, a deeper understanding of the role of the nonadditive exchange‐correlation functional and its implications for local bond formation is critically needed.

In this work, we report a systematic study analyzing the suitability of PBET for application on metal surfaces toward efficient catalyst screening. Therefore, we analyze the binding energy and electronic structure of a series of CO_2_ reduction intermediates on a Cu(111) surface. The surface is described by cluster models. To get insights into the different error sources and to understand their origin, we focus on DFT‐in‐DFT embedding, where the active subsystem is treated using a hybrid exchange‐correlation functional (PBE0),^[^ ^84^ ^]^ while the environment is described on the GGA level (PBE).^[^ ^85^ ^]^ Both functionals differ only in the fraction of exact HF exchange and allow us to unravel the impact of the nonadditive exchange functional systematically. Based on this, we demonstrate that the nonadditive exchange functional is the primary contributor to the energetic errors in the PBET method when applied to metal clusters. By increasing the fraction of the exact HF exchange to match the one used on the active system, we significantly enhance the accuracy. Furthermore, we reveal that this adjustment is essential for achieving improved accuracy as the size of the active system increases. The systematic improvement with the size of the active subsystem is a key property for embedding methods as it ensures systematic improvability.

We further discuss the influence of the choice of the active orbital space on the accuracy and present a guideline for consistent and reliable active space selection along a reaction pathway based on the SPADE algorithm.

PBET provides a simple framework for QM‐in‐QM embedding. However, its value for metallic catalysts has not systematically been investigated and might be challenged on conceptual grounds due to the inherent problem of partitioning a system with nonlocal correlations. In this work, however, we demonstrate that these challenges can be overcome and PBET is a powerful tool to study heterogeneous (electro‐)catalysis for large numbers of surface structures due to the reduction in computational cost enabled by the partitioning. Our study hence provides a thorough basis for future developments in WFT‐in‐DFT embedding.

## Results and Discussion

### PBET Applied on CO@Cu(111)‐Clusters

**Figure 1:**
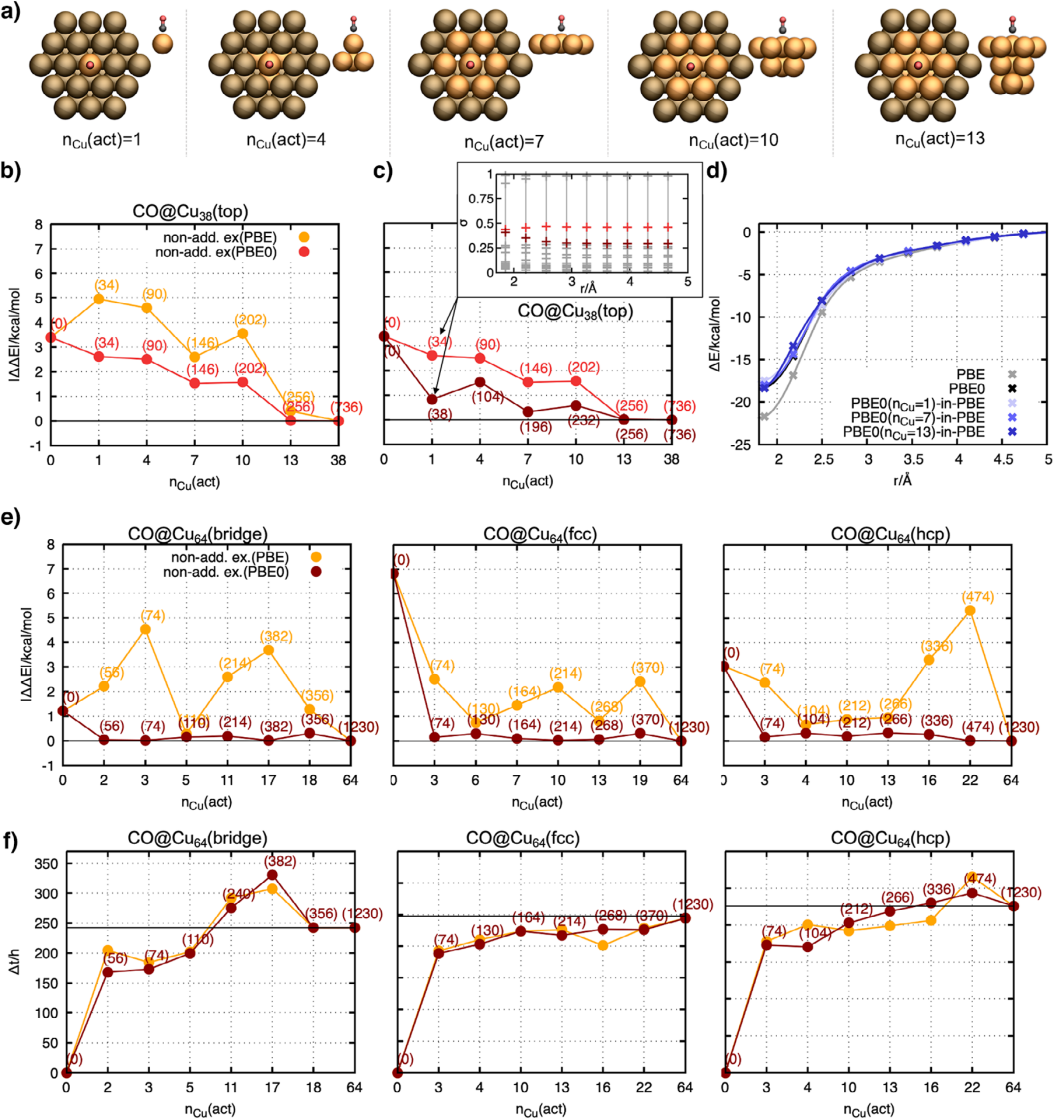
a) Ball‐and‐stick representation of the Cu_38_–cluster model used to model the dissociation curve of CO adsorbed in the top position. From left to right, the size of the active subsystem increases. The active subsystem includes the CO molecule plus *n*_Cu_ (act) copper atoms. The active copper atoms are highlighted in orange. b) Error of PBET obtained binding energies of CO@Cu_38_ with CO adsorbed in the top position w.r.t. a calculation employing PBE0 for the entire system. The red curve corresponds to the evaluation of the nonadditive exchange‐correlation with the PBE0 functional, while the orange curve corresponds to the evaluation with the PBE functional. c) Variation of the active molecular orbital space in PBET. Both curves are evaluated with the nonadditive PBE0 exchange‐correlation functional. The inset shows the hierarchy of the corresponding singular values σ along the reaction coordinate. The colored markers (light and dark red) correspond to singular values of the molecular orbitals and indicate where the molecular orbital space is partitioned. d) Selected dissociation curves highlighting the accuracy of the embedding approach. All curves are based on the nonadditive PBE0 exchange‐correlation functional. e) Error of the binding energies calculated with PBET with respect to the binding energy calculated fully with the PBE0 functional for different adsorption sites on different Cu clusters. f) CPU time needed for the optimization of the electronic structure. The values are calculated with respect to a pure PBE calculation, providing the initial guess for the molecular orbitals for the PBE0 as well as the embedding calculations. Both curves (with PBE (orange) and PBE0 (dark red) nonadditive exchange‐correlation) consider the same active molecular orbital space.

We chose CO adsorbed on Cu(111) as our initial test system. Since the surface binding site is represented by a cluster model, this model must be large enough to reproduce the binding energy calculated using periodic DFT as accurately as possible and also allow for a systematic variation of the active cluster size. Therefore, depending on the adsorption site of the CO molecule on the Cu(111) surface, different cluster models are used (see Figure S2). For CO adsorbed in the top position, a Cu_38_–cluster as shown in Figure 1a was selected. Since the CO molecule binds directly to one Cu atom from the surface, the smallest active subsystem considers only CO and the Cu atom to which it binds. Based on this, the size of the active subsystem can systematically be increased by assigning the next neighbor atoms from the first, second, and finally third layer to the active subsystem (see Figures 1a and S2). The size of the active subsystem is given by the number of Cu atoms assigned to the active subsystem (*n*_Cu_(act) ∈ {1,4,7,10,13}). With this Cu_38_–cluster, the error in the binding energy with respect to the periodic plane‐wave reference calculation is around 4.7 kcal mol^−1^. To assess the accuracy of the PBET approach, the binding energy is also calculated using the PBE0 functional and the same DEF2‐TZVP basis set^[^ ^86^ ^]^ used in all calculations (see Supporting Information for further computational details). Note that the influence factors, such as surrounding water or other solvents, as well as intermolecular interactions, which are important for realistic modeling,^[^ ^87^, ^88^, ^89^ ^]^ are not included in this study. These contributions do not interfere with the embedding scheme. Therefore, we focus here only on idealized models that allow us to develop a consistent and accurate embedding approach that can be verified with meaningful theory‐to‐theory comparisons. Avoiding the use of dispersion corrections allows us to analyze all errors purely as a function of the PBET approach and the density functionals used. Employing structure‐dependent dispersion corrections such as D3^[^ ^90^, ^91^ ^]^ would result in a constant term for all calculations and hence not influence the reported errors, whereas density‐dependent contributions such as D4^[^ ^92^ ^]^ are expected to deviate only to a minor extent for the different computational setups. To compare to experimental values, however, inclusion of a dispersion correction is crucial.^[^ ^89^ ^]^ Within the embedding framework, the best result can be achieved when the entire cluster model is active, which is equivalent to an SCF calculation purely on the PBE0 level. The binding energy obtained by employing PBET is calculated for all the different active cluster sizes. The error in the binding energy with respect to the full PBE0 calculation is visualized in Figure 1b. They are computed once using PBE as the nonadditive exchange‐correlation functional (orange curve) and once using PBE0 (red curve). The selected active molecular orbital spaces between both curves are chosen to be identical for all *n*_Cu_(act) such that all differences originate from the choice of the nonadditive exchange functional. Clearly, no convergence with the size of the active subsystem but large fluctuations are observed when PBE is selected as the nonadditive exchange‐correlation functional. In contrast, the PBE0 functional results in significantly lower and near‐monotonous convergence to the full PBE0 limit with increasing active system sizes. In Figure 1c, we analyze the impact of the active space size. Since the deviations obtained with the PBE0 functional for the nonadditive exchange‐correlation were significantly smaller, we start from these results and vary only the active molecular orbital space size. As desired, increasing the active molecular orbital space increases the accuracy for all active cluster sizes considered. The most remarkable correction is shown for the smallest CO@Cu_1_‐in‐Cu_37_ (*n*_Cu_(act) = 1) system. Although the active molecular orbital space is only extended by four additional molecular orbitals, an additional energy correction of 1.8 kcal mol^−1^ has been achieved, pushing the total error with respect to the full PBE0 calculation below 1 kcal mol^−1^. To explain this observation, the evolution of the singular value distributions along the reaction trajectory is visualized in the inset of Figure 1c. All molecular orbitals with singular values higher than the light or dark red highlighted singular values, respectively, are treated as active. The singular values highlighted in dark‐red change strongly with the structure, indicating strong changes of the corresponding molecular orbital along the dissociation pathway. This is a clear indication that this molecular orbital contributes to the CO─Cu bond formation‐/breaking process. Therefore, it must be included in the active molecular orbital space in order to calculate the binding energy accurately. The light red‐curve illustrates the reduced accuracy if this crucial molecular orbital is excluded from the active space.

From this analysis, we have two main observations: First, best accuracy was achieved, when treating the nonadditive exchange‐correlation functional at the higher level of theory. Second, the active molecular orbital space size must be selected such that all molecular orbitals that contribute significantly to bond formation/‐breaking process are included. To highlight the quality achieved following these rules, Figure 1d shows the full dissociation curves corresponding to the data points with active subsystems of size $n_{\mathrm{Cu}}\left(\mathrm{act}\right)\in \left\{1,7,13\right\}$ from the dark‐red curve in Figure 1c. The active subsystems are defined by two characteristic quantities: i) the active cluster size, which is given by the number of Cu atoms manually selected to constitute the active subsystem, and ii) the size of the active molecular orbitals space. The active molecular orbital space is determined based on the SPADE algorithm.^[^ ^62^, ^76^ ^]^ This procedure assigns a singular value to each occupied molecular orbital, which indicates its importance with respect to the selected active cluster geometry (for more information about the active orbital space selection see Suppoting Information and Figure S1). Even for the smallest active cluster size, the PBET curves are almost indistinguishable from the full PBE0 reference. To further benchmark this approach, we calculated the binding energies of CO on different adsorption sites (bridge, fcc, and hcp) for both choices of the nonadditive exchange‐correlation functional (PBE and PBE0) for several active cluster sizes (see Figure S3). The results are displayed in Figure 1e. The cluster models used here contain 64 Cu atoms in total (see Figure S2). In line with the results for CO in the top position, the error in the binding energy fluctuates strongly if PBE is used for the nonadditive exchange‐correlation functional, and no systematic convergence is observed for any of the adsorption sites considered here. Contrary to this, the errors calculated based on the nonadditive PBE0 functional converge even for small active cluster sizes rapidly below 0.5 kcal mol^−1^. Further, our analysis reveals that the accuracy gained by evaluating the nonadditive exchange‐correlation with the PBE0 functional does not significantly increase the computational cost in terms of computation time and memory compared to the PBE functional (see Figures 1f and S4). That both treatments of the nonadditive exchange‐correlation functional lead to comparable numerical costs relies on the fact that the density of the supersystem is only evaluated once with the nonadditive exchange‐correlation functional, in order to construct the embedding potential acting on the active subsystem. Any additional efforts involved in this individual evaluation step are negligible compared to the costs of the overall relaxation of the molecular orbitals of the active subsystem. However, a significant reduction in computational time is only achieved for small active cluster sizes (*n*_Cu_ ≤ 5). Memory use for all embedding calculations is reduced compared to a full PBE0 calculation.

In summary, this analysis demonstrates the significant potential of the PBET approach, as it enables us to achieve nearly PBE0 accuracy, although only a small fraction of the full system is treated at this higher level of theory.

### Impact of the Nonadditive Exchange Functional

**Figure 2:**
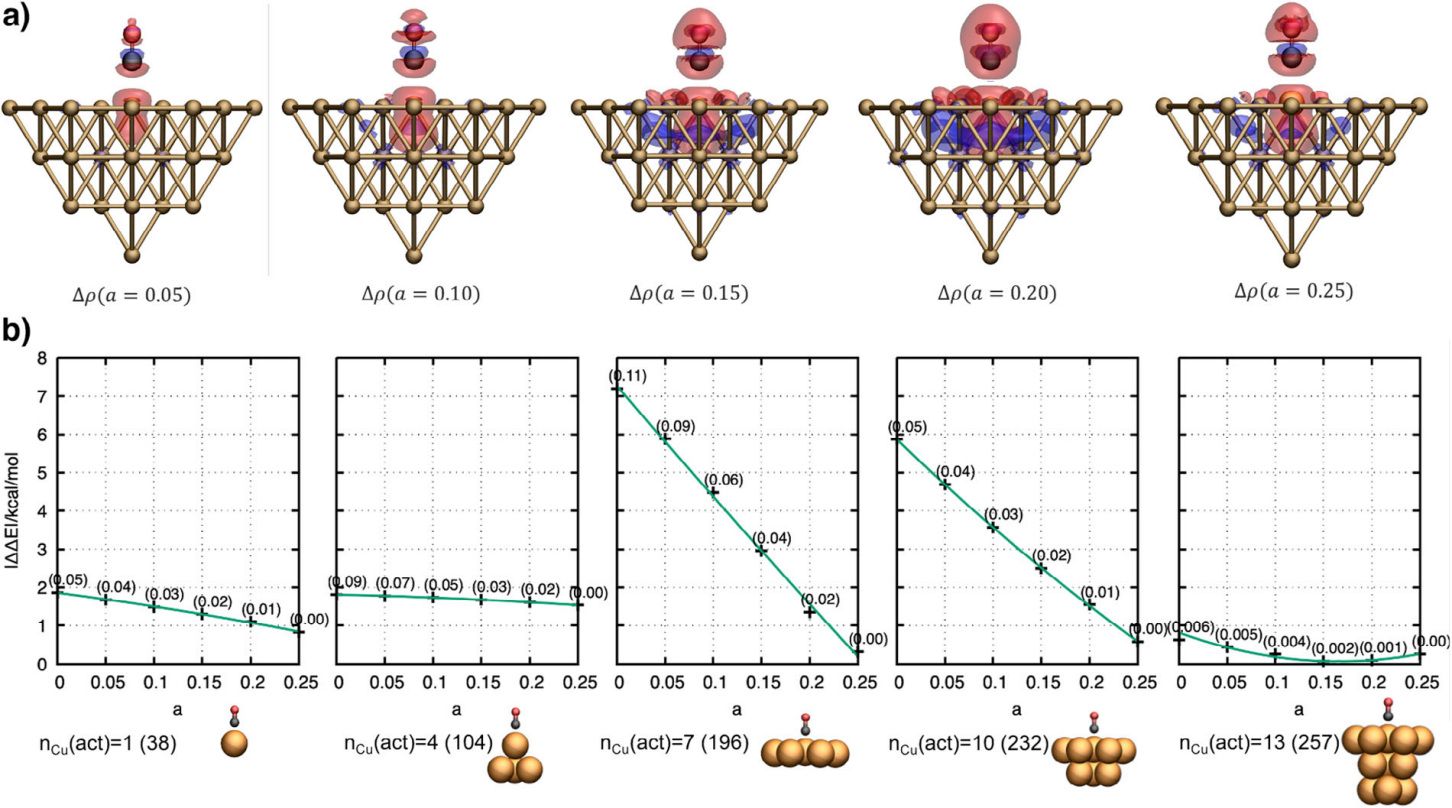
Impact of the fraction of exact Hartree–Fock exchange on the nonadditive exchange‐correlation functional. The parameter *a* indicates the fraction of exact HF exchange. a) Density differences of CO@Cu_1_‐in‐Cu_37_ calculated according to Δρ(*a*)_adsorbed_ = ρ(*a*)_adsorbed_ − ρ(PBE)_adsorbed_), where ρ(*a*)_adsorbed_ is the density of the active system calculated for a given value *a*. b) Absolute error in the binding energy with respect to the fraction of exact HF exchange (*a*). The active cluster size (*n*_Cu_ (act)) is shown below the diagrams. The numbers in parentheses denote the number of active molecular orbitals used for the embedding calculation. The thin green lines are only to guide the eye, highlighting the trend of the curves. The numbers above the data points in parentheses represent the Mulliken charge differences on the active molecular orbitals corresponding to CO@Cu_1_ with respect to the embedding calculation with *a*  =  0.25.

As discussed above, the choice of the nonadditive exchange functional has a strong impact on the error introduced in PBET when applied to reactions on metal surfaces. To develop a deeper understanding of the reason for this, the differences in the electron density, calculated with PBE and PBE0 for the nonadditive exchange contribution, are computed and shown in Figure 2a. For this analysis, the CO@Cu_1_‐in‐Cu_37_ cluster model is used as a compact, yet representative example. We note that PBE and PBE0 differ only in the fraction of the exact exchange. Varying the fraction of exact exchange (denoted by *a*) according to $E^{\mathrm{non}-\mathrm{add}.\;\mathrm{ex}\;}\left(a\right)=E_{C}^{\mathrm{PBE}}+\left(1-a\right)E_{X}^{\mathrm{PBE}}+{\mathrm{aE}}_{X}^{\mathrm{exact}\;\mathrm{HF}}$ allows us to study the impact on the electron density systematically. The electron density differences shown in Figure 2a are calculated for increasing fractions of exact exchange, all with respect to PBE (*a*  =  0). A fraction of *a*  =  0.25 corresponds to the PBE0 functional. In all diagrams, the red areas represent charge depletion zones, while the blue areas represent charge accumulation zones. With increasing exact exchange in the nonadditive exchange‐correlation functional, two key features emerge: i) The charge density around the local C─O bond localizes, and ii) the electron density on the active subsystem is reduced and pushed toward the nonactive (or environmental) part of the cluster, which decreases the total charges on the active molecular orbitals of the active subsystem. This observation is also supported by Figure 2b), where the error of the binding energy is calculated for all different active subsystems ($n_{\mathrm{Cu}}\left(\mathrm{act}\right)\in$ {1,4,7,10,13}) and decreases mostly linearly with increasing fractions of exact exchange. Only for the largest active cluster considered (*n*_Cu_(act) = 13), the curve is parabolic rather than linear, but here, all calculated errors are below 0.5 kcal mol^−1^. Note that the discrepancies of the slope steepness for different definitions of the active subsystems are likely a result of the different active cluster shapes. Although the active subsystem is systematically extended by increasing the number of active Cu atoms stepwise, each extension changes the symmetry of the active system and correspondingly the interaction with the environment. Moreover, we also observe an almost linear decrease of the effective charge around the locally formed bond (described by the subsystem CO─Cu) with an increasing fraction of exact exchange, which indicates a reduction of the delocalization error on the localized active subsystem.^[^ ^80^ ^]^ The charges are calculated based on the atomic Mulliken charges of CO and the Cu atom to which it binds.

In order to highlight the importance of mitigating delocalization errors and to show that a mixture of exact exchange promotes this effect, we have validated further combinations of exchange correlation functionals for the respective subsystems, all indicating that a fraction of exact exchange improves the accuracy and ensures convergence with increasing active cluster size (see Figure S5).

### Application of PBET to Open‐Shell Adsorbates

**Figure 3:**
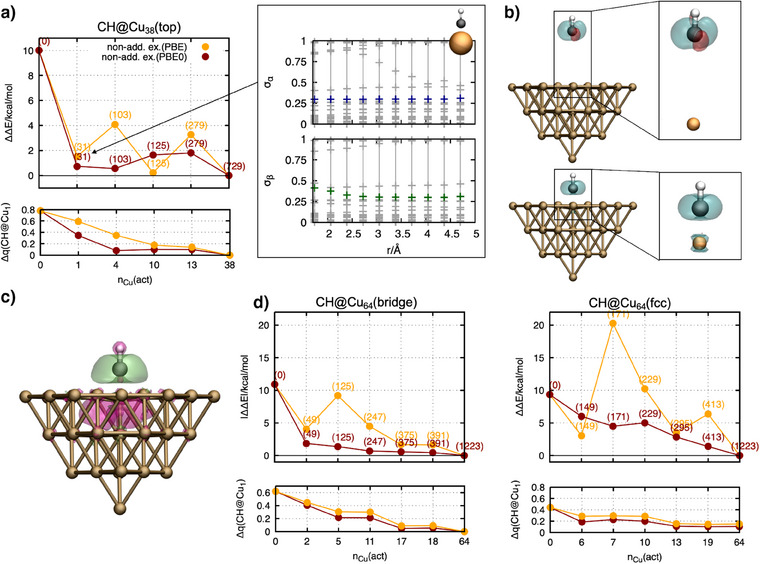
PBET for open‐shell systems. CH is used as a representative adsorbate. a) The top panel shows the error of the PBET binding energies for CH in the top position on a Cu_38_–cluster with respect to the binding energy calculated fully with the PBE0 functional. The numbers in parentheses indicate the size of the active molecular orbital space. The diagrams on the right‐hand side show the evolution of the singular value distribution of the CH@Cu_1_ active subsystem molecular orbitals along the reaction coordinate for the alpha and beta electrons. The bottom panel shows the change in the local charges on the CH@Cu_1_ subsystem calculated with respect to the full PBE0 calculation. b) Spin densities ρ_α/β_ (blue areas represent the population of alpha electrons, and red areas the population of beta electrons) of CH@Cu_1_‐in‐Cu_37_ for CH being dissociated (top panel) and adsorbed on the surface (bottom panel). For this calculation, PBE0 is used for the nonadditive exchange functional. c) Visualization of the deviation of spin densities calculated using PBE0 and PBE for the nonadditive exchange functional (ΔΔρ_spin_ = ρ_spin_(PBE0) − ρ_spin_(PBE), with Δρ_spin_ = Δρ_α_ − Δρ_β_). Green areas indicate positive and pink areas indicate negative values. The illustration shows thus stronger localization of the unpaired electron, when using the PBE0 nonadditive exchange functional. d) Error of the PBET binding energies of CH in bridge and fcc position for Cu_64_ clusters with respect to the binding energy calculated fully on PBE0 level.

We have demonstrated that the delocalization error on the active subsystem is effectively reduced when evaluating the nonadditive exchange‐correlation functional at the higher level of theory, or, more precisely, when a certain fraction of exact exchange is included. This leads to a more accurate description of the local intermolecular interaction and, thus, of the respective binding energies for adsorbates on metallic systems. Building on this foundation, we now analyze open‐shell active subsystems, which present additional challenges for PBET. Open‐shell adsorbates, with their unpaired electrons, are inherently more sensitive to delocalization errors due to the delocalization tendencies of the remaining spin imposed by approximate exchange functionals.^[^ ^93^, ^94^, ^95^ ^]^ Furthermore, in PBET, where the system is partitioned into active and environmental subsystems, it remains uncertain whether small active clusters can adequately capture the transfer of the spin density from the adsorbate to the metal cluster. Insufficient treatment of this process can compromise the accuracy of the method. Therefore, we aim to investigate whether PBET can address the challenges posed by open‐shell systems. By taking CH as a representative open‐shell adsorbate, we will analyze whether small active clusters are sufficient for modeling the CH─Cu interactions and if delocalization errors can also effectively be reduced by treating the nonadditive exchange‐correlation functional at the PBE0 level. The binding energy of CH in the top position is around *E*_bind_(CH)  =  87.9 kcal mol^−1^ (obtained from periodic DFT calculations employing the PBE functional), much higher than the closed‐shell CO probe molecule analyzed before ($E_{\mathrm{bind}}\left(\mathrm{CO}\right)$ = 16.8 kcal mol^−1^, calculated with the same approach. Based on the same Cu_38_–cluster model as used before for the CO─Cu_38_ system, the calculated PBET CH binding energy with the PBE functional is around *E*_bind_ = 68.1 kcal mol^−1^. Carrying out the supersystem calculation fully with the PBE0 functional reduces the binding energy by around 10 kcal mol^−1^ to *E*_bind_ = 58.1 kcal mol^−1^. Additionally, we calculated the effective charge around the local CH─Cu bond. In contrast to the adsorbed carbon monoxide, where the difference between full PBE and PBE0 was approximately 0.08 e, the charge difference for CH was found to be 0.78 e and thus significantly larger. This indicates that the exact‐exchange in PBE0 pushes electron density efficiently to the cluster, while PBE promotes the delocalization over the entire system, which leads to a strong overstabilization of the locally formed bond when using PBE, associated with a more pronounced delocalization error. Acknowledging this, the question arises if PBET can reduce the delocalization error by introducing charge transfer even for small active cluster sizes. However, when performing the PBET calculation, the first prominent phenomenon observed is the significant changes of the singular values along the dissociation trajectory (see Figure 3a, right panel). Due to the strong interaction of the unpaired spin with the metal surface, the corresponding molecular orbital changes strongly when breaking the bond by increasing the distance. To describe the C─Cu bond sufficiently, these strongly changing molecular orbitals must be included in the active molecular subsystem, exactly as discussed for the previous examples. Regarding the choice of the nonadditive exchange‐correlation functional, a similar behavior as for the closed‐shell system is observed for PBE and PBE0. While the energy corresponding to the nonadditive PBE exchange functional fluctuates strongly with increasing system (*n*_Cu_(act)) size, the error corresponding to the PBE0 nonadditive exchange functional reduces quickly to below 2 kcal mol^−1^, already for the smallest active cluster sizes of *n*_Cu_(act) = 1 (see Figure 3a, top left panel) for this challenging case. The overall error reduction of PBET by more than 8 kcal mol^−1^ compared to the pure PBE calculation when treating the nonadditive exchange functional with the PBE0 functional, along with the stronger reduction in effective charges under the same treatment (see Figure 3a, bottom left panel), highlights appropriate correction of the delocalization error. Figure 3b shows the spin density calculated from PBET evaluating the nonadditive exchange functional with the PBE0 functional for the dissociated CH molecule (upper picture) and for the adsorbed CH molecule (bottom picture). While the unpaired spin is localized completely on the CH molecule when being apart from the surface, the bond‐forming process transfers and localizes spin density to the bonded Cu atom. However, comparing the spin densities obtained with PBE0 as nonadditive exchange functional with PBE reveals stronger localization in the case of the PBE0 functional (see Figure 3c). At the same time, we have calculated stronger charge transfer to the environmental cluster. Both characteristics together indicate a correction of the introduced delocalization error that comes from the nonadditive PBE functional, which spuriously distributes spin density around the bonded Cu atom. To further benchmark these results, we calculated the error of the binding energies of CH adsorbed on different adsorption sites (bridge and hcp) (see Figure 3d). In agreement with the results for the top position, PBET successfully reduces the error in the binding energies when using PBE0 as nonadditive exchange functional.

### Binding Energies of CO_2_ Reaction Intermediates

**Figure 4:**
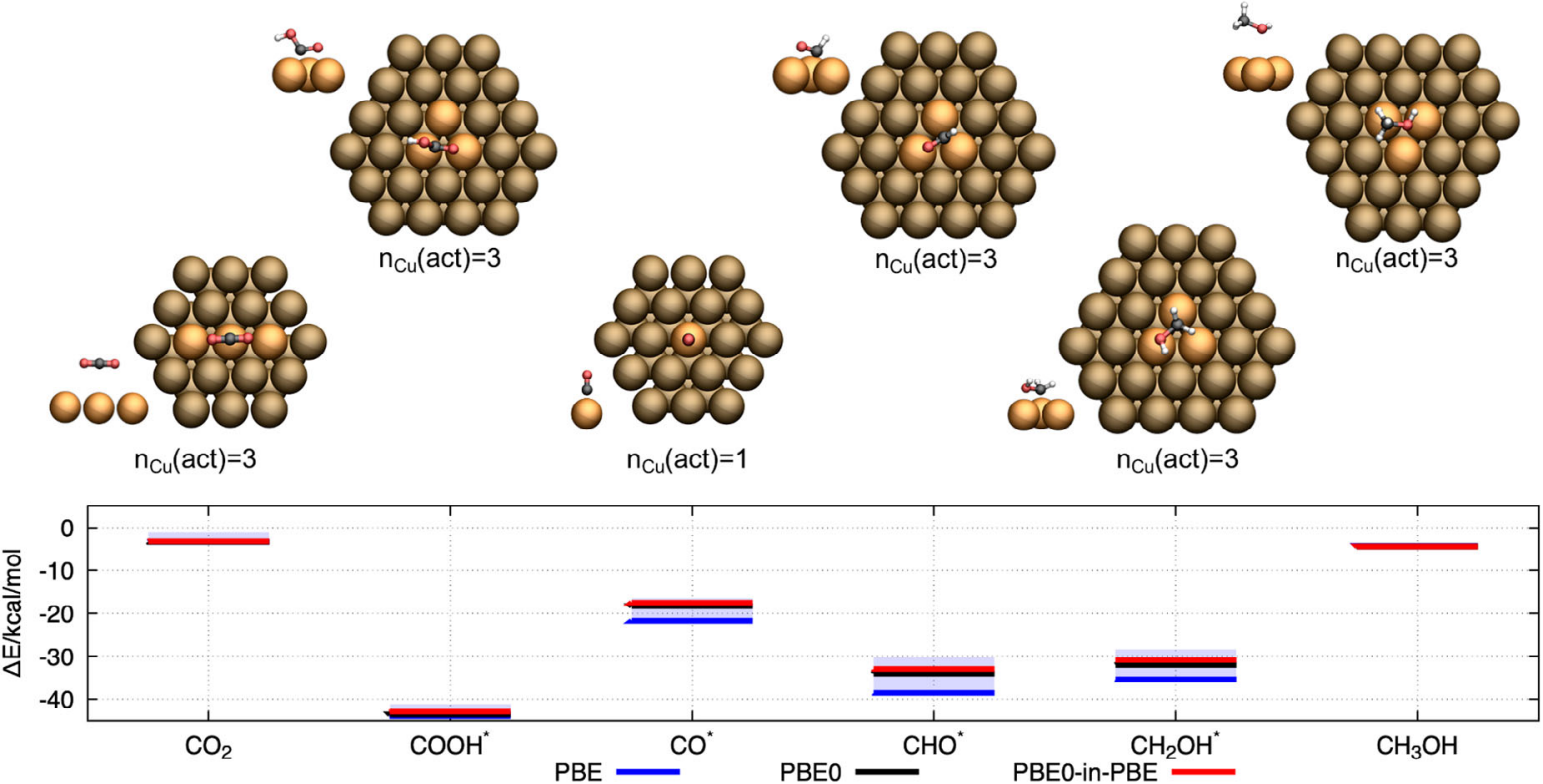
Binding energies of CO_2_ reaction intermediates, considering a pathway to CH_3_OH. The binding energies are calculated purely on the PBE level (blue lines), purely on the PBE0 level (black lines), and by using the PBET (red lines). The respective cluster structures used are depicted above the diagram. The active subsystem is defined by the interaction site on the surface, and the active Cu atoms are indicated in orange. The Cu atoms highlighted in brown are assigned to the environmental subsystem. The blue shadows above the PBE energies indicate the deviation from the plane‐wave results obtained with VASP using the PBE functional and considering periodic boundary conditions.

This work highlights the applicability of PBET to study the binding energies of adsorbates on metal surfaces with a focus on CO_2_ reduction reaction intermediates as important prototypical cases. For a broader benchmarking and to confirm the identified requirements on the active orbital space selection and regarding the choice of the nonadditive exchange functional, we calculated the binding energies of several CO_2_ reduction intermediates: CO_2_, COOH*, CO*, COH*, CH_2_OH*, and CH_3_OH. The cluster models used in these calculations depend on the adsorption site to which the reaction intermediate binds. The same clusters as previously introduced for calculating binding energies in the top, bridge, fcc, and hcp positions are considered here (see Figures 4 and S2, S3). Further, we choose only the smallest active cluster sizes ($n_{\mathrm{Cu}}\left(\mathrm{act}\right)\in \left\{1,3\right\}$), in order to evaluate the accuracy for the most efficient PBET settings. The active orbital space is selected such that all strongly changing molecular orbitals along the dissociation curve are assigned to the active subsystem. The nonadditive exchange functional is evaluated using the PBE0 functional. In addition to the PBET calculations, we will compute the binding energy using a plane‐wave approach as reference. These calculations are conducted using the PBE exchange functional. For further comparison, the cluster calculations are also performed with pure PBE and PBE0 functionals without embedding. The results are shown in Figure 4. In all calculations, where the molecule adsorbed at the surface, the differences between PBET and full PBE0 calculations are below 2 kcal mol^−1^, with even lower errors for closed‐shell species. Therefore, this study reveals that PBET is capable to predict the binding energies of CO_2_ reaction intermediates efficiently and accurately. Despite this success, this analysis also reveals one remaining problem when using PBET to study interaction energies on metal surfaces. The error that is introduced by selecting a cluster model instead of using periodic boundary conditions is, in some calculations, larger than the error corrected by using PBET. As the focus of this study is the evaluation of an accurate setup for PBET applied to metallic systems, the same cluster geometry has been used for all calculations (with respect to the adsorption site). Future work to mitigate this effect will focus on guidelines for choosing adequate cluster models. Two conceivable solutions are i) to replace the embedding potential used for the calculation of the active subsystem by one obtained from cost‐effective (potentially semi‐empirical) periodic calculations, or ii) to explicitly account for the finite band gap inherent to cluster models by preparing the electronic structure in a bonding state.^[^ ^32^, ^35^ ^]^

## Conclusion

In conclusion, we presented a systematic study analyzing the accuracy of the projection‐based embedding theory when applied to study the binding energy of adsorbates on metal surfaces represented by cluster models. This work highlights the importance of an appropriate choice for the nonadditive exchange functional when treating metal surfaces, which has not been reported previously. We have shown that the choice of the nonadditive exchange functional strongly impacts the electron density distribution in the active subsystem. Although the active subsystem is described at a higher level of theory, the delocalization of molecular orbitals on the active subsystem is promoted by the embedding potential when treating the nonadditive exchange functional at the GGA level, thus promoting delocalization errors. Based on this, we demonstrated that the results fluctuate strongly and nonmonotonically with respect to the selected active cluster sizes. In contrast, mixing fractions of exact exchange to the nonadditive exchange functional substantially reduces the delocalization error for the active subsystem, leading to improved accuracy and to smooth convergence of the binding energy with respect to the active cluster size. These observations hold for both closed‐ and open‐shell adsorbates, where in the latter delocalization errors are typically more pronounced.

This study highlights the importance of mitigating delocalization errors on the active subsystem. However, we demonstrate this effect only for PBE0‐in‐PBE embedding by mixing varying amounts of exact HF exchange in the nonadditive exchange functional. For embedding calculations with different functionals or for the application of WFT‐in‐DFT embedding, extensive benchmark studies are needed in order to elucidate the best combination of exchange‐correlation descriptions for the respective subsystems and subsystem interactions.

Regarding a consistent selection of the active molecular orbital space, this study recommends tracing the evolution of the singular values along the dissociation curve, as originally proposed by our recently developed ACE‐of‐SPADE algorithm. Based on this, the active molecular orbital space must be defined such that all molecular orbitals that change along the reaction trajectory are assigned to the active system.

This study shows how PBET can be successfully applied to study chemical reactions on metal surfaces represented by cluster models. While in this approach, an important prerequisite is the definition of adequate cluster models, which must accurately reproduce the plane‐wave results at the lower level of theory, calculating the embedding potential from periodic calculations is also conceivable.^[^ ^63^, ^96^ ^]^

When the guidelines outlined in this study are followed, PBET is an adequate and systematically improvable tool for the computational research of heterogeneous (electro‐)catalysis and surface chemistry. The reduction of computational cost that is achieved by focusing the computational effort on a small region of interest paves the way for efficient yet accurate catalyst screening. In future work, we will aim to further increase the accuracy of such simulations by incorporating WF‐in‐DFT embedding approaches.

## Supporting Information

Computational details, tabulated binding energies, visualization of the cluster models and additional benchmarking data.

## Conflict of Interests

The authors declare no conflict of interest.

## Supporting information



Supporting Information

## Data Availability

The data that support the findings of this study are available in the Supporting Information of this article.
