# Supplementary material for: Efficient Electronic‐Structure Methods Toward Catalyst Screening: Projection‐Based Embedding Theory for CO2 Reduction Reaction Intermediates
Source: Angew Chem Int Ed Engl. 2025 Aug 7;64(38):e202503418. doi: 10.1002/anie.202503418 (PMC12435423; doi:10.1002/anie.202503418)
Supplement: Supplementary file 1 — Supporting Information [file ANIE-64-e202503418-s001.pdf]

Supporting Information

Efficient Electronic-Structure Methods Toward Catalyst Screening: Projection-Based Embedding Theory for CO2 Reduction Reaction Intermediates

Elena Kolodzeiski[a] and *Christopher J. Stein*[a,b,c]

[a)] Department of Chemistry, TUM School of Natural Sciences Technical University of Munich, Garching, Germany

[b)] Catalysis Research Center, Technical University of Munich, Garching, Germany

[c)] Atomistic Modeling Center, Technical University of Munich, Garching, Germany

Contents

1. **Computational Details**
2. **Orbital Localization with SPADE**
3. **Binding Energies**
4. **Cluster Models**
5. **Memory Demands**
6. **Benchmarking further Combinations of Exchange-Correlation Functionals**
7. **References**

1. Computational Details

Before the respective embedding calculations, structure optimizations were carried out with periodic DFT. These calculations were performed with the Vienna ab Initio Simulation Package (VASP 5.4) using the PBE functional and projected augmented wave (PAW) pseudo-potentials.[1,2] The plane-wave energies were limited by an energy cut-off of 400 eV. The electronic states were described through Gaussian smearing with a width of 0.2 eV. The convergence threshold for the forces acting on the nuclei was set to 0.02 eV/Å, and the electronic relaxation criterion was set to 10^{-7} eV. For the Brillouin zone integration, a 6x6x1 k-point grid was applied. The surface is modeled by a unit cell containing three 5x5 Cu-layers with a lattice constant of 3.65 Å. The CO_2 reaction intermediates studied in this work are adsorbed on the surface. To avoid spurious self-interaction between periodic images in *z*-direction, a 40 Å vacuum layer was considered. During the structure optimizations, the upper two layers and the adsorbed molecule were fully relaxed, while the nuclear coordinates of the bottom Cu-layer was fixed to the bulk values. The relaxed structures are used as a basis for tailoring the cluster models (see Figure S1). The bare cluster models are designed such that they exhibit a closed-shell ground state. All cluster calculations are conducted using a modified version of the Q-Chem electronic-structure program package[3] with the DEF2-TZVP basis set.[4] The modification in Q-Chem allows us to vary the functional describing the non-additive exchange during the PBET calculations. The Stuttgart/Cologne effective core potential ECP10MDF was used for the Cu atoms.[5] Further, density fitting for evaluating the Coulomb and exchange integrals based on the auxiliary basis set RIJK-def2-TZVPP was used.[6] To verify the accuracy of the embedding approach, for each CO_2 reaction intermediate, a supersystem calculation is conducted fully with both PBE and PBE0. The solution obtained from the supersystem calculation with the PBE functional provides an appropriate initial guess for the PBE0 supersystem calculation as well as for the subsequent PBET calculations. The binding energies obtained with the PBE0 functional represent the target accuracy. It should be noted that in this study, large cluster sizes (Cu_{38} and Cu_{64}) are used, as the cluster models are supposed to represent the surface properties of a bulk structure sufficiently (see Figure S1). However, large cluster sizes lead to complicated potential energy surfaces with many local minima. This is a challenge for the self-consistent field (SCF) optimization, resulting potentially in different local minima for slightly different structures, making even the determination of the spin ground-

state challenging. This holds particularly for unrestricted calculations on open-shell systems. Therefore, the optimization parameters, such as the initial guess in the PBE supersystem calculation or the optimizer (DIIS and GDM),[7-9] were chosen manually since no setting ensured convergence in all cases. Further, if convergence problems prevail, the maximum overlap method has been applied to guide the system to a specific local minimum. Using the converged PBE wavefunction from the supersystem calculation as an initial guess for PBET ensures that all PBET calculations converge to the same local minima on the potential energy surface.

The orbital partitioning during the PBET calculations is conducted with a slight modification to our recently developed ACE-of-SPADE algorithm, which allows us to track the evolution of the molecular orbitals along the trajectory. The orbital space selection is semi-automatically conducted based on the evolution of the singular values along the reaction pathway such that all molecular orbitals whose singular values change strongly along a given trajectory are included. The active orbital space must be chosen large enough to accommodate all those orbitals. As the bare cluster model is designed such that its unbiased electronic structure converges to a closed-shell singlet, only orbital space partitioning, which retains the closed-shell character of the environmental subsystem, is considered.

The dissociation pathway is constructed by varying the Cu-substrate bond length up to a maximum distance of 5 Å. The state, where the molecule is 5 Å apart from the surface is defined as the dissociated state. Further structures along the reaction trajectory are for each substrate and each adsorption site individually defined by interpolation between the initial and final steps, considering 10 intermediate steps. In the case of CH, the cluster geometry is affected due to the strong bond, lifting the bonded Cu-atoms slightly. This effect is compensated with structural linear interpolation.

Note, errors arising from the finite band gap of the embedded cluster models (active subsystem $E_A^{\text{high/low}}$) cancel out due to the ONIOM-like energy expression of the projection-based embedding theory:

$$\begin{aligned} E_{\text{tot}}^{\text{ONIOM}} &= E_A^{\text{high}} - E_A^{\text{low}} + E_{\text{total}}^{\text{low}} \\ &= E_A^{\text{high}} - E_A^{\text{low}} + \underbrace{E_A^{\text{low}} + E_B^{\text{low}} + E_{\text{A-B interaction}}^{\text{low}}}_{E_{\text{total}}^{\text{low}}} \\ &= E_A^{\text{high}} + E_B^{\text{low}} + E_{\text{A-B interaction}}^{\text{low}} = E_{\text{tot}}^{\text{Projection-based Emb.}} \end{aligned}$$

2. Orbital Localization with SPADE

The active space partitioning is based on two steps: i) the assignment of atoms belonging to the active system and ii) the assignment of the molecular orbitals corresponding to the respective subsystems defined in i). While the former step is straightforward and depends only on the choice of the user, the assignment of the molecular orbitals constitutes a bigger challenge, as particularly for delocalized molecular orbitals, it is not clear which molecular orbitals belong to the active and which molecular orbitals belong to the environmental subsystem. In order to conduct this assignment, we use the SPADE algorithm. This algorithm projects the molecular orbitals onto an orthogonalized atomic orbital basis of the active subsystem. Based on this, a singular value decomposition (SVD) is performed on the active subsystem coefficient matrix. The derived transformation matrices from the SVD allow the rotation of the canonical molecular orbitals to a set of localized SPADE orbitals. Each SPADE orbital is characterized by a singular value, which indicates its occupation on the active subsystem.[10]

Further, when energy differences along a reaction pathway are to be calculated, it is important that all calculations for all the different geometries are based on the same set of molecular orbitals in order to avoid inconsistencies. This procedure is particularly for metal systems, which exhibit a large number of delocalized (near-) degenerate molecular orbitals, which is very challenging. In this regard, we have developed the ACE-of-SPADE algorithm, which allows us to track the evolution of the molecular orbitals along the trajectory based on the evolution of the corresponding singular value distributions for all geometries (see Figure S1).[11] The singular values highlighted in red indicate each reaction coordinate by its value and importance to the active system. Large changes along the trajectory indicate that the molecular orbital changes in response to the bond-forming or breaking process, indicating participation in this process. The last molecular in this spectrum that changes the most along the trajectory is the last molecular orbital assigned to the active system. All molecular orbitals with singular values larger than this threshold are treated as active, too.

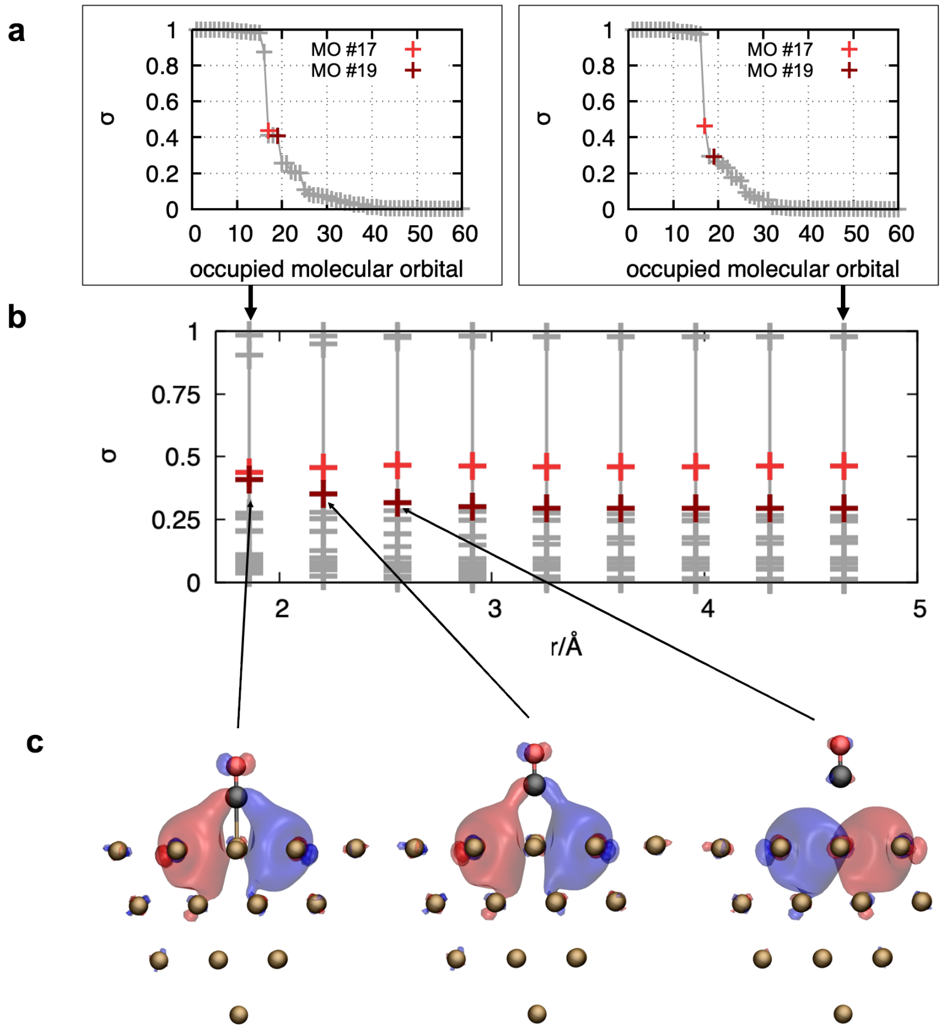

Figure S1. a) Singular value distribution of at reaction coordinate r=1.86 Å and r=4.74 Å for CO on Cu111 in top position. b) Evolution of the singular values distribution along the reaction coordinate r. c) Visualization of the 38th molecular orbital and how it changes from r=1.86 Å to r=2.50 Å. The change of the molecular orbital is indicated by the change of the corresponding singular value highlighted in dark-red in b).

3. Binding Energies

	periodic DFT(PBE)	Cluster (PBE)	Cluster (PBE0)
CO*(top)	-20.06	-21.72	-18.33
CO*(bridge)	-21.76	-20.29	-13.55
CO*(fcc)	-24.36	-25.91	-14.53
CO*(hcp)	-24.11	-22.12	-19.08

Table S1. Binding energies of CO adsorbed on the Cu(111) surface in kcal/mol. The columns indicate the different models (plane-wave and cluster) as well as the different exchange functionals (PBE and PBE0). The rows label the different adsorption sites.

	periodic DFT(PBE)	Cluster (PBE)	Cluster (PBE0)
CH*(top)	-87.93	-68.08	-58.08
CH*(bridge)	-134.65	-105.72	-94.83
CH*(fcc)	-137.50	-104.74	-95.39

Table S2. Binding energies of CH adsorbed on the Cu(111) surface in kcal/mol. The columns indicate the different models (plane-wave and cluster) as well as the different exchange functionals (PBE and PBE0). The rows label the different adsorption sites.

	periodic DFT(PBE)	Cluster (PBE)	Cluster (PBE0)
CO2*	0.08	-3.39	-3.31
COOH*	-47.05	-43.93	-43.62
CHO*	-35.55	-38.53	-34.06
CH_2OH*	-32.07	-35.34	-32.08
CH_3OH*	-1.95	-4.34	-4.44

Table S3. Binding energies of different CO_2 reaction intermediates adsorbed on the Cu(111) surface in kcal/mol. The columns indicate the different models (plane-wave and cluster) and different exchange functionals (PBE and PBE0). The rows label the different reaction intermediates.

4. Cluster Models

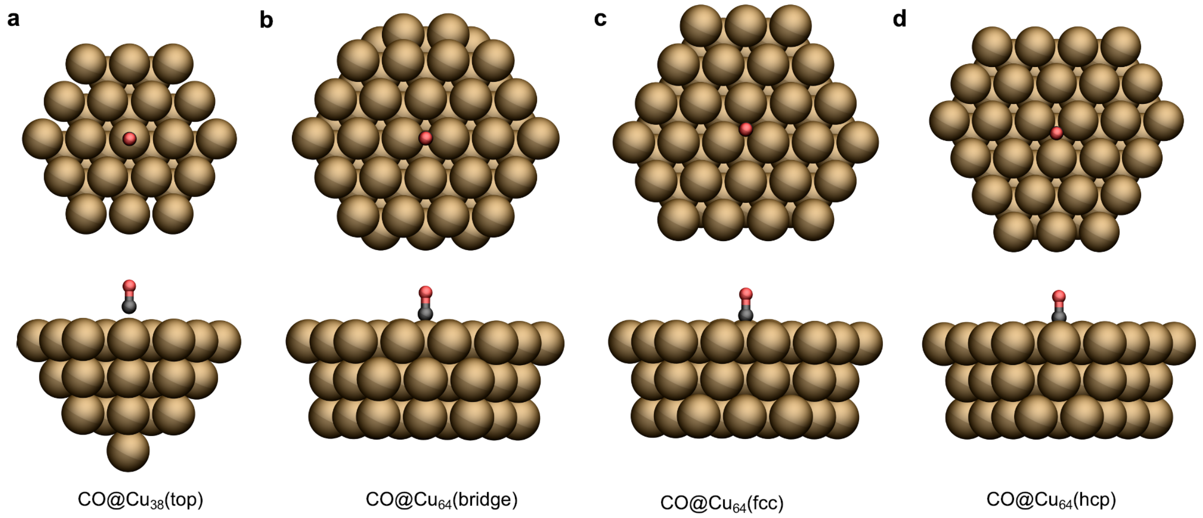

Figure S2. Ball-and-stick representation of the cluster models used in the main manuscript. Panel a) shows CO and the respective cluster model when adsorbed in a top position, b) belongs to an adsorption in bridge position, c) shows adsorption in the fcc-hollow position, and d) adsorption in the hcp-hollow position. The number of Cu-atoms included in the respective models are indicted below.

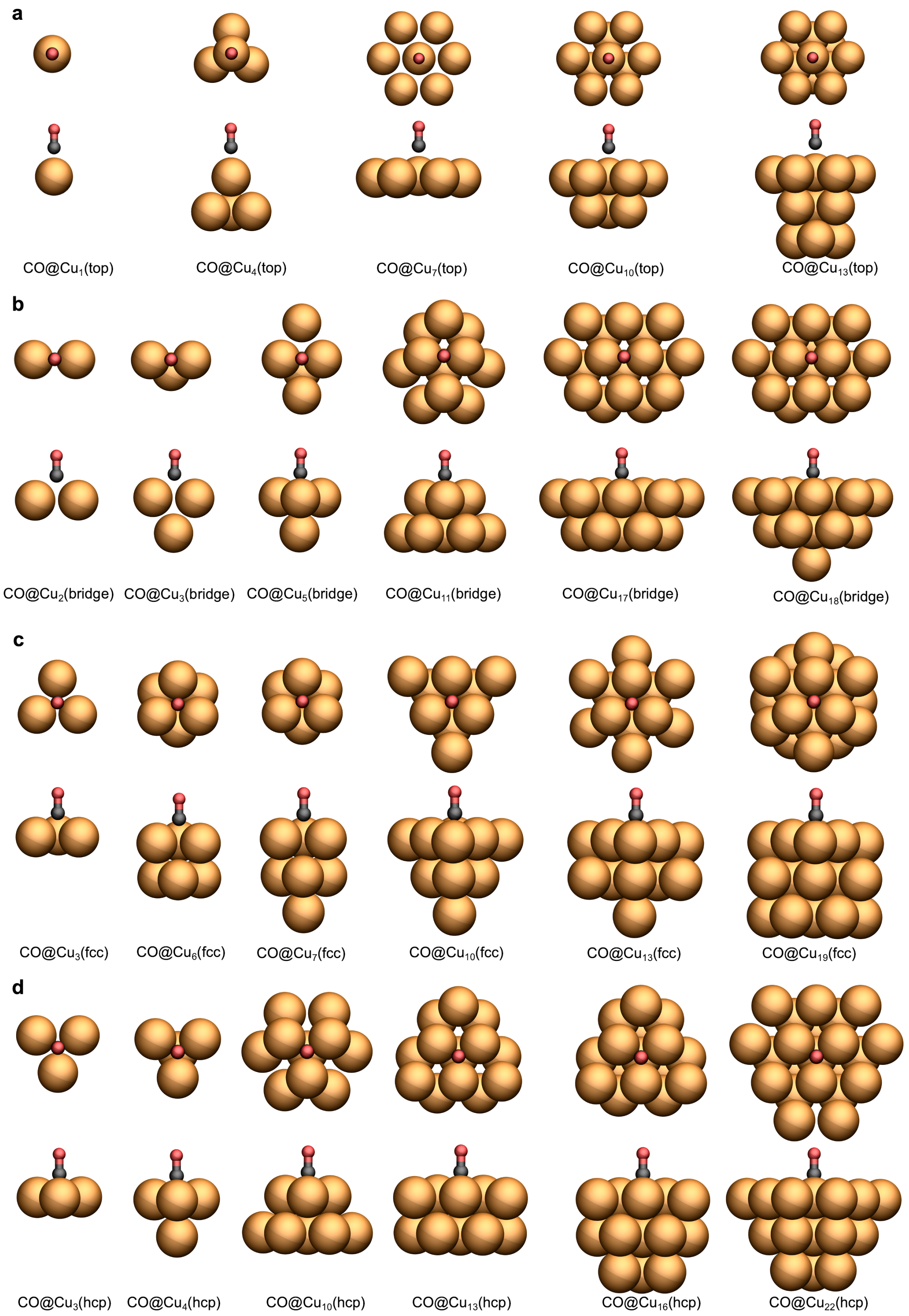

Figure S3. Ball-and-Stick representation of the active clusters used in the main manuscript for describing substrate adsorption in top position (a), bridge position (b), fcc position (c) and hcp position (d). CO is used as probe molecule highlighting the adsorption site. The active cluster size is indicated below the respective panels.

5. Memory Demands

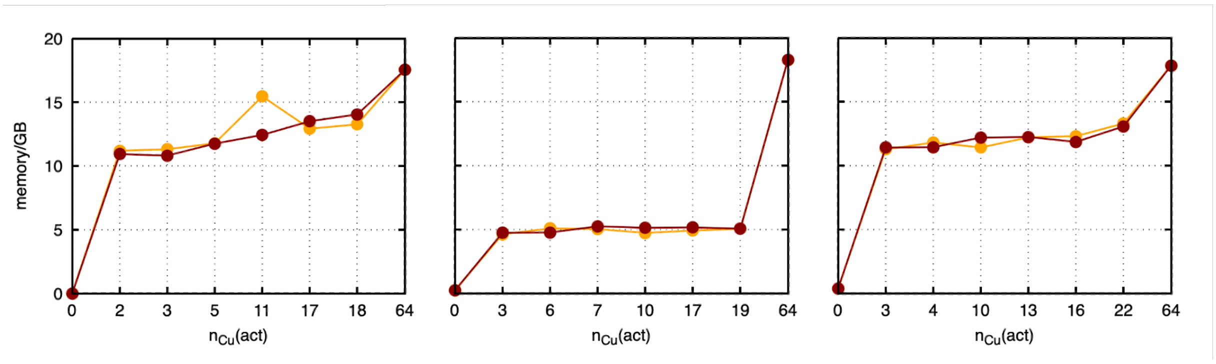

Figure S4. Memory needed for the optimization of the electronic structure of CO in bridge (a), fcc (b) and hcp (c) position. The values are calculated with respect to a pure PBE calculation, providing the initial guess for the molecular orbitals for the PBE0 as well as the embedding calculations.

6. Benchmarking further Combinations of Exchange-Correlation Functionals

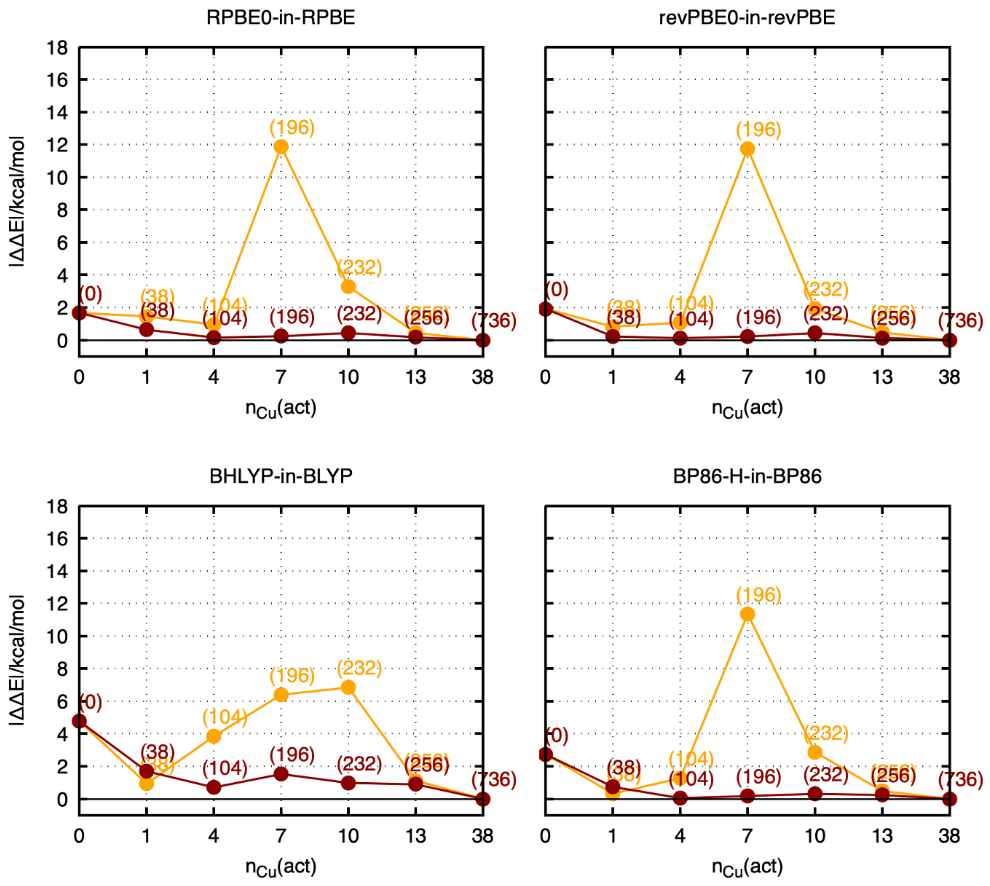

Figure S5. Error of PBET obtained binding energies of CO@Cu_{38} with CO adsorbed in the top position w.r.t. a calculation employing the higher level of theory for the entire system for different combinations of exchange functionals. The red curve corresponds to the evaluation of the non-additive exchange-correlation with the

respective hybrid functional, while the orange curve corresponds to the evaluation with the GGA functional. The BP86-H hybrid exchange functional considers 25% exact exchange.

7. References

(1) Kresse, G.; Furthmüller, J. Efficiency of ab-initio total energy calculations for metals and semiconductors using a plane-wave basis set. *Comput. Mater. Sci.* **1996**, *6* (1), 15-50.
(2) Kresse, G.; Furthmüller, J. Efficient iterative schemes for ab initio total-energy calculations using a plane-wave basis set. *Phys. Rev. B* **1996**, *54* (16), 11169-11186.
(3) Epifanovsky, E.; Gilbert, A. T. B.; Feng, X.; Lee, J.; Mao, Y.; Mardirossian, N.; Pokhilko, P.; White, A. F.; Coons, M. P.; Dempwolff, A. L.; Gan, Z.; Hait, D.; Horn, P. R.; Jacobson, L. D.; Kaliman, I.; Kussmann, J.; Lange, A. W.; Lao, K. U.; Levine, D. S.; Liu, J.; McKenzie, S. C.; Morrison, A. F.; Nanda, K. D.; Plasser, F.; Rehn, D. R.; Vidal, M. L.; You, Z.-Q.; Zhu, Y.; Alam, B.; Albrecht, B. J.; Aldossary, A.; Alguire, E.; Andersen, J. H.; Athavale, V.; Barton, D.; Begam, K.; Behn, A.; Bellonzi, N.; Bernard, Y. A.; Berquist, E. J.; Burton, H. G. A.; Carreras, A.; Carter-Fenk, K.; Chakraborty, R.; Chien, A. D.; Closser, K. D.; Cofer-Shabica, V.; Dasgupta, S.; de Wergifosse, M.; Deng, J.; Diedenhofen, M.; Do, H.; Ehlert, S.; Fang, P.-T.; Fatehi, S.; Feng, Q.; Friedhoff, T.; Gayvert, J.; Ge, Q.; Gidofalvi, G.; Goldey, M.; Gomes, J.; González-Espinoza, C. E.; Gulania, S.; Gunina, A. O.; Hanson-Heine, M. W. D.; Harbach, P. H. P.; Hauser, A.; Herbst, M. F.; Hernández Vera, M.; Hodecker, M.; Holden, Z. C.; Houck, S.; Huang, X.; Hui, K.; Huynh, B. C.; Ivanov, M.; Jász, Á.; Ji, H.; Jiang, H.; Kaduk, B.; Kähler, S.; Khistyaev, K.; Kim, J.; Kis, G.; Klunzinger, P.; Koczor-Benda, Z.; Koh, J. H.; Kosenkov, D.; Koulias, L.; Kowalczyk, T.; Krauter, C. M.; Kue, K.; Kunitsa, A.; Kus, T.; Ladjánszki, I.; Landau, A.; Lawler, K. V.; Lefrancois, D.; Lehtola, S.; Li, R. R.; Li, Y.-P.; Liang, J.; Liebenthal, M.; Lin, H.-H.; Lin, Y.-S.; Liu, F.; Liu, K.-Y.; Loipersberger, M.; Luenser, A.; Manjanath, A.; Manohar, P.; Mansoor, E.; Manzer, S. F.; Mao, S.-P.; Marenich, A. V.; Markovich, T.; Mason, S.; Maurer, S. A.; McLaughlin, P. F.; Menger, M. F. S. J.; Mewes, J.-M.; Mewes, S. A.; Morgante, P.; Mullinax, J. W.; Oosterbaan, K. J.; Paran, G.; Paul, A. C.; Paul, S. K.; Pavošević, F.; Pei, Z.; Prager, S.; Proynov, E. I.; Rák, Á.; Ramos-Cordoba, E.; Rana, B.; Rask, A. E.; Rettig, A.; Richard, R. M.; Rob, F.; Rossomme, E.; Scheele, T.; Scheurer, M.; Schneider, M.; Sergueev, N.; Sharada, S. M.; Skomorowski, W.; Small, D. W.; Stein, C. J.; Su, Y.-C.; Sundstrom, E. J.; Tao, Z.; Thirman, J.; Tornai, G. J.; Tsuchimochi, T.; Tubman, N. M.; Veccham, S. P.; Vydrov, O.; Wenzel, J.; Witte, J.; Yamada, A.; Yao, K.; Yeganeh, S.; Yost, S. R.; Zech, A.; Zhang, I. Y.; Zhang, X.; Zhang, Y.; Zuev, D.; Aspuru-Guzik, A.; Bell, A. T.; Besley, N. A.; Bravaya, K. B.; Brooks, B. R.; Casanova, D.; Chai, J.-D.; Coriani, S.; Cramer, C. J.; Cserey, G.; DePrince, A. E., III; DiStasio, R. A., Jr.; Dreuw, A.; Dunietz, B. D.; Furlani, T. R.; Goddard, W. A., III; Hammes-Schiffer, S.; Head-Gordon, T.; Hehre, W. J.; Hsu, C.-P.; Jagau, T.-C.; Jung, Y.; Klamt, A.; Kong, J.; Lambrecht, D. S.; Liang, W.; Mayhall, N. J.; McCurdy, C. W.; Neaton, J. B.; Ochsenfeld, C.; Parkhill, J. A.; Peverati, R.; Rassolov, V. A.; Shao, Y.; Slipchenko, L. V.; Stauch, T.; Steele, R. P.; Subotnik, J. E.; Thom, A. J. W.; Tkatchenko, A.; Truhlar, D. G.; Van Voorhis, T.; Wesolowski, T. A.; Whaley, K. B.; Woodcock, H. L., III; Zimmerman, P. M.; Faraji, S.; Gill, P. M. W.; Head-Gordon, M.; Herbert, J. M.; Krylov, A. I. Software for the frontiers of quantum chemistry: An overview of developments in the Q-Chem 5 package. *Chem. Phys.* **2021**, *155* (8), 084801.

(4) Weigend, F.; Ahlrichs, R. Balanced basis sets of split valence, triple zeta valence and quadruple zeta valence quality for H to Rn: Design and assessment of accuracy. *Phys. Chem. Chem. Phys.* **2005**, *7* (18), 3297-3305.
(5) Figgen, D.; Rauhut, G.; Dolg, M.; Stoll, H. Energy-consistent pseudopotentials for group 11 and 12 atoms: adjustment to multi-configuration Dirac–Hartree–Fock data. *Chem. Phys.* **2005**, *311* (1), 227-244.
(6) Weigend, F. Hartree–Fock exchange fitting basis sets for H to Rn. *J. Comput. Chem.* **2008**, *29* (2), 167-175.
(7) Van Voorhis, T.; Head-Gordon, M. A geometric approach to direct minimization. *Mol. Phys.* **2002**, *100* (11), 1713-1721.
(8) Pulay, P. Convergence acceleration of iterative sequences. the case of scf iteration. *Chem. Phys. Lett.* **1980**, *73* (2), 393-398.
(9) Pulay, P. Improved SCF convergence acceleration. *J. Comput. Chem.* **1982**, *3* (4), 556-560.
(10) Claudino, D.; Mayhall, N. J. Automatic Partition of Orbital Spaces Based on Singular Value Decomposition in the Context of Embedding Theories. *J. Chem. Theory Comput.* **2019**, *15* (2), 1053-1064.
(11) Kolodzeiski, E.; Stein, C. J. Automated, Consistent, and Even-Handed Selection of Active Orbital Spaces for Quantum Embedding. *J. Chem. Theory Comput.* **2023**, *19* (19), 6643-6655.
